# Resource Allocation in Downlink VLC-NOMA Systems for Factory Automation Scenario

**DOI:** 10.3390/s22239407

**Published:** 2022-12-02

**Authors:** Won-Jae Ryu, Jae-Woo Kim, Dong-Seong Kim

**Affiliations:** 1 ICT Convergence Research Center, Kumoh National Institute of Technology, Gumi 39177, Gyeongbuk, Republic of Korea; 2 Department of IT Convergence Engineering, Kumoh National Institute of Technology, Gumi 39177, Gyeongbuk, Republic of Korea

**Keywords:** visible light communication (VLC), non-orthogonal multiple access (NOMA), reliability, power allocation, deep neural network (DNN), user-pairing scheme, factory automation

## Abstract

Industry 4.0 requires high-speed data exchange that includes fast, reliable, low-latency, and cost-effective data transmissions. As visible light communication (VLC) can provide reliable, low-latency, and secure connections that do not penetrate walls and are immune to electromagnetic interference; it can be considered a solution for Industry 4.0. The non-orthogonal multiple access (NOMA) technique can achieve high spectral efficiency using the same frequency and time resources for multiple users. It means that smaller amounts of resources will be used compared with orthogonal multiple access (OMA). Therefore, handling multiple data transmissions with VLC-NOMA can be easier for factory automation than OMA. However, as the transmit power is split, the reliability is reduced. Therefore, this study proposed a deep neural network (DNN)-based power-allocation algorithm (DBPA) to improve the reliability of the system. Further, to schedule multiple nodes in VLC-NOMA system, a priority-based user-pairing (PBUP) scheme is proposed. The proposed techniques in VLC-NOMA system were evaluated in terms of the factory automation scenario and showed that it improves reliability and reduces missed deadlines.

## 1. Introduction

Advancements in light emitting diode (LED) technology have caused LEDs to have long life cycles and to overheat less during operation, making them more cost- and energy-efficient. This has resulted in their large-scale use. LEDs are not only used as a light source but can also be used as transmitters for visible light communication (VLC) [1]. As VLC can provide high speeds and security, it can be an appropriate solution for indoor wireless communications [2]. VLC can be a solution to overcome the shortage of radio frequency resources, as it adopts an unlicensed spectrum. As such, the potential of VLC can be advantageous for industrial wireless applications. There have also been considerations to apply VLC to industrial wireless communications [3].

As non-orthogonal multiple access (NOMA) can achieve high spectral efficiency by adopting superposition coding (SC), it can simultaneously transmit signals to both the near-user (NU) and the far-user (FU) using the same frequency resource [4]. Therefore, NOMA was proposed for VLC [5,6,7,8].

The signal for FU can be canceled by successive interference cancellation (SIC); then, the signal for NU can be decoded [9]. Instead, as the signal for FU is with the highest power allocation, the signal can be decoded without SIC. VLC-NOMA systems were evaluated in [7,10] in terms of their coverage probability and ergodic sum rate. Kizilirmak et al. evaluated the performance of VLC-NOMA compared with orthogonal frequency-division multiple access in terms of data rate [11]. Marshoud et al. evaluated the bit error rate (BER) of a NOMA system made up of a user pair including three or more users [12].

In this study, we describe the VLC-NOMA system applied to factory automation, proposing a deep neural network (DNN)-based power allocation scheme and a priority-based user pairing (PBUP) scheme. As mentioned above, VLC adopts unlicensed bandwidth, is free from radio frequency interference, and has secure transmission by signals not penetrating walls [8]. Therefore, the advantages of VLC can be considered solutions for factory automation. For more additions, factory automation should handle periodic transmission for each node [13]. Therefore, handling periodic transmission should be scheduled, and the usage of resources for scheduling should be reduced to avoid missed deadlines of real-time transmission. Adopting NOMA can reduce the usage of resources, as NOMA can transmit multiple signals within the same resource.

We derived the BER performance of both users in a user pair, in terms of both perfect and imperfect SICs, and showed a balanced power ratio that considers both NU and FU in terms of reliability. Reliability is a key performance indicator for defining the stability and accuracy of a system [14]. To improve reliability, DNN-based power allocation (DBPA) was proposed with the derived BER performance.

Previous studies have applied DNNs to VLC. Lee et al. proposed a deep learning framework for the design of an on–off keying (OOK)-based binary signaling transceiver in dimmable VLC systems [15]. Ulkar et al. suggested a VLC module that uses a DNN to reduce the complexity caused by handling both the error rate and level of illumination simultaneously [16]. However, these studies did not consider NOMA. Although Lin et al. suggested a signal DNN-based demodulator in a VLC-NOMA system [17], their study applied a DNN to a demodulator rather than a power allocation algorithm. In contrast, the proposed DBPA in this study focuses on finding the minimum point of average BER to improve reliability for both users.

In addition, there have been studies for resource allocation in NOMA systems with radio frequency. Ali et al. suggested a power allocation algorithm using DNN in a device-to-device NOMA system to maximize sum capacity [18]. Kumaresan et al. studied user-clustering and power allocation in downlink NOMA systems to maximize throughput [19]. Fu et al. suggested dynamic power control for NOMA transmissions to minimize the transmission delay with the consideration of each user’s transmission deadline and the total power constraint [20]. Manglayev et al. proposed machine-learning- and deep-learning-based power allocation schemes that find near-optimal solutions with regard to sum capacity and provide low computational costs in NOMA systems [21]. As mentioned in the above studies, DNNs can be used for resource allocation for wireless communication systems due to the low computational complexity [22].

Moreover, VLC is considered for industrial applications [3]. There have been reliability issues for industrial applications [23,24]. Therefore, in this study, we proposed DBPA and PBUP as solutions to improve reliability and real-time transmission.

Even though the VLC-NOMA system can provide efficient transmissions to reduce the usage of resources, the decoding error is larger than that of the VLC orthogonal multiple access (OMA) system. The VLC-OMA allocates the whole transmit power for one data transmission in a resource. Instead, the VLC-NOMA system transmits superposed signals splitting transmit power; then, the decoding error is larger than that of the VLC orthogonal multiple access (OMA) systems. It can cause significant problems for factory automation. Therefore, the reliability of the VLC-NOMA system should be improved. DBPA can be considered a solution to cover the decoding error caused by splitting transmit power. In this study, DBPA is proposed to improve the reliability of the VLC-NOMA system for factory automation. Moreover, the proposed VLC-NOMA system should handle two signals at a timeslot.

As the VLC-NOMA system should handle a maximum of two signals in a timeslot, each signal for each node should be paired. Instead, the VLC-OMA system has to only schedule transmitting orders in timeslots. However, the VLC-NOMA system should pair two nodes before scheduling. Therefore, in this study, PBUP is considered a way to pair two nodes based on transmission priority.

The contributions of this study are as follows:

- Evaluating the reliability of the VLC-NOMA system in terms of the perfect and imperfect SICs. It provides the simulation and numerical results in terms of BER.
- Proposing VLC-NOMA system for a factory automation scenario to solve missed deadlines caused by a lack of resources.
- Proposing DBPA to solve the degradation of reliability caused by adopting the VLC-NOMA system.
- Proposing PBUP to pair two nodes based on priority and channel state for the VLC-NOMA system.

The remainder of this paper is structured as follows: The system model is described in Section 2; Section 3 proposes DBPA and PBUP; Section 4 explains the evaluation and analysis of the downlink VLC-NOMA system in terms of reliability and of DBPA compared with other power allocation algorithms, and evaluates PBUP in a factory automation scenario; Section 5 concludes the paper and describes future work.

## 2. System Model

This study is on the VLC-NOMA system for a factory automation scenario. Therefore, the first subsection is the system model for the VLC-NOMA system and the second subsection is the scenario of factory automation.

### 2.1. The VLC-NOMA System

In this subsection, a downlink VLC-NOMA system is described for the BER performance. The system is made up of a single LED as a transmitter, and two users with photodiodes (PDs) as receivers in an NOMA user pair in an indoor environment. The modulation technique adopts OOK, and the OOK signals for NU and FU are superposed for transmission from the transmitter.

The line-of-sight VLC channel model was averaged between the LED and users [25]. The channel model is expressed as follows:

(1) $$h=P\frac{\left(m+1\right)cos^{m}\left(\Phi \right)}{2\pi d^{2}}AT\left(\phi \right)g\left(\phi \right)cos\left(\phi \right),$$

(2) $$m=\frac{-ln(2)}{ln(cos\left(\Phi_{1/2}\right))}.$$

The notations of the above equations are described in Table 1. The channel gain between the LED and a user can be calculated by (1).

**Figure 1:**
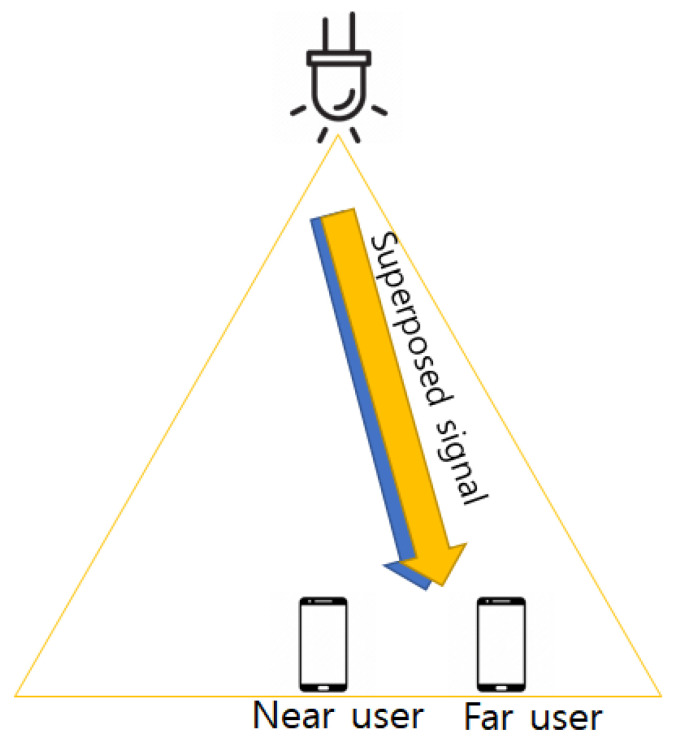
Downlink VLC-NOMA system.

NOMA superimposes and transmits signals to users. As described in Figure 1, the system is made up of one LED as a transmitter and two users, including NU and FU. An equation for it is as follows:

(3) $$y=h\left(x_{n}+p_{f}x_{f}\right)+n_{o}.$$

As the system superimposes signals, NU signal $x_{n}$ and FU signal $x_{f}$ are superimposed.

The signal for NU is regarded as interference for FU. Therefore, the signal $y_{f}$ is as follows:

(4) $$y_{f}=h_{f}p_{f}x_{f}+\underset{\mathrm{Interference}}{\underset{︸}{h_{f}p_{n}x_{n}}}+n_{o}.$$

The signal $y_{n}$ for NU after SIC is as follows:

(5) $$y_{n}=h_{n}p_{n}x_{n}+n_{o}.$$

As the signal for FU is canceled by SIC, only the other signal for NU is residual and then decoded by NU. However, in cases where SIC fails, decoding the signal is hard for NU. Therefore, the success ratio of decoding signals to FU affects the success of SIC.

To calculate the error mentioned above, the BER equations for both users are going to be dealt with. There are four cases for bits of NU and FU using OOK in the downlink VLC-NOMA system.

(6) $$p\left(0,0\right)=\frac{1}{4},\;p\left(0,1\right)=\frac{1}{4},\;p\left(1,0\right)=\frac{1}{4},\;p\left(1,1\right)=\frac{1}{4},$$

where p(x,y) denotes the probability of bits, *x* for FU, and *y* for NU.

**Figure 2:**
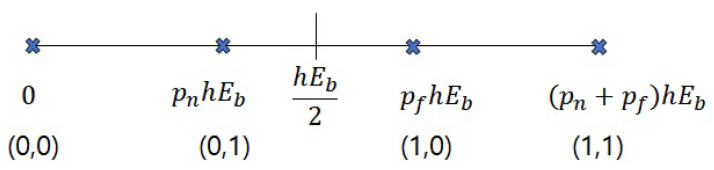
The constellation for the received signal.

The constellation for the received signal is described in Figure 2. We assume $p_{n}+p_{f}=1$. In the case of received signals, the constellation of the receiver is affected by channel gain *h*. Therefore, the larger *h* is, the lower BER is. The threshold for signals for FU is $\frac{hE_{b}}{2}$, because FU should decode the signal regardless of the existence of the signal for NU, as shown in Figure 2. This means that irrespective of whether the bit for NU is 0 or 1, the range of signals for FU starts at the same point, $\frac{hE_{b}}{2}$.

Therefore, the BER for FU, where *x* is 1 and *y* is 0, is as below:

(7) $$p\left(e|1,0\right)=\frac{1}{\sqrt{\pi N_{0}}}\int_{-\infty }^{\frac{h\sqrt{E_{b}}}{2}}e^{\frac{-{\left(y-p_{f}h\sqrt{E_{b}}\right)}^{2}}{N_{0}}}dy.$$

$E_{b}$ means energy per bit; $N_{0}$ is noise power; and as the bit for FU is 1 and the bit for NU is 0, the signal for (7) will be converged at $P_{f}h\sqrt{E_{b}}$ in the constellation shown in Figure 2. The reason why $p_{n}$ was not expressed in (7) is that the bit 0 for NU means no power in OOK. When the bit is 0, no power is allocated for transmitting the signal.

Continue to derive (7):

(8) $$p\left(e|1,0\right)=\frac{1}{\sqrt{\pi }}\int_{-\infty }^{\frac{\frac{h\sqrt{E_{b}}}{2}-p_{f}h\sqrt{E_{b}}}{\sqrt{N_{0}}}}e^{-z^{2}}dz,$$

(9) $$p\left(e|1,0\right)=\frac{1}{\sqrt{\pi }}\int_{\left(p_{f}-\frac{1}{2}\right)h\sqrt{\frac{E_{b}}{N_{0}}}}^{\infty }e^{-z^{2}}dz.$$

Therefore,

(10) $$p\left(e|1,0\right)=\frac{1}{2}erfc\left(\left(p_{f}-\frac{1}{2}\right)h\sqrt{\frac{E_{b}}{N_{0}}}\right).$$

As shown in Figure 2, the boundary of the signal for FU is from $\frac{hE_{b}}{2}$ regardless of the signal for NU. This means that the threshold for FU will not be changed by the existence of signals for NU. Then, as $p_{n}+p_{f}=1$, the cases $p_{n}+p_{f}$, which are p(e|1,1) and p(e|0,0), are considered as simple OOK BERs. As drawn in Figure 2, the decision threshold is $\frac{hE_{b}}{2}$. Therefore, the equation for the cases p(e|1,1) and p(e|0,0) is as follows:

(11) $$p\left(e|1,1\right)=p\left(e|0,0\right)=\frac{1}{2}erfc\left(0.5h\sqrt{\frac{E_{b}}{N_{0}}}\right).$$

Therefore,

(12) $$\begin{array}{c} p(e)=p(0,0)p(e|0,0)+p(0,1)p(e|0,1)\\ +p(1,0)p(e|1,0)+p(1,1)p(e|1,1), \end{array}$$

(13) $$\begin{array}{c} p(e|0,1)=p(e|0,1)\\ p(e|0,0)=p(e|1,1), \end{array}$$

(14) $$p\left(e\right)=\frac{1}{4}\left(erfc\left(0.5h\sqrt{\frac{E_{b}}{N_{0}}}\right)+erfc\left(\left(p_{f}-\frac{1}{2}\right)h\sqrt{\frac{E_{b}}{N_{0}}}\right)\right).$$

Finally, the BER for FU is calculated by (14).

Next, the BER for NU must be calculated. First, perfect SIC is assumed. The BER equation simply adds the power ratio for NU into a simple OOK BER equation. The BER equation for an NU with a perfect SIC is as follows:

(15) $$p\;=\;\frac{1}{2}erfc\left(\frac{p_{n}h}{2}\sqrt{\frac{E_{b}}{N_{0}}}\right).$$

Based on (14) and (15), the BER equation for an NU with imperfect SIC can be derived. There are two cases of error: one wherein SIC succeeds and decoding fails, and the other wherein both SIC and decoding fail. The first case is as follows:

(16) $$\begin{array}{c} p\left(e|SIC\right)=\left[1-\frac{1}{4}\left(erfc\left(0.5h\sqrt{\frac{E_{b}}{N_{0}}}\right)+erfc\left(\left(p_{f}-\frac{1}{2}\right)h\sqrt{\frac{E_{b}}{N_{0}}}\right)\right)\right]\times \frac{1}{2}erfc\left(\frac{p_{n}h}{2}\sqrt{\frac{E_{b}}{N_{0}}}\right)\\ =\left[0.5-\frac{1}{8}\left(erfc\left(0.5h\sqrt{\frac{E_{b}}{N_{0}}}\right)+erfc\left(\left(p_{f}-\frac{1}{2}\right)h\sqrt{\frac{E_{b}}{N_{0}}}\right)\right)\right]erfc\left(\frac{p_{n}h}{2}\sqrt{\frac{E_{b}}{N_{0}}}\right) \end{array}$$

The second case is as follows:

(17) $$\begin{array}{c} p\left(e|failedSIC\right)=\frac{1}{4}\left(erfc\left(0.5h\sqrt{\frac{E_{b}}{N_{0}}}\right)+erfc\left(\left(p_{f}-\frac{1}{2}\right)h\sqrt{\frac{E_{b}}{N_{0}}}\right)\right)-[p\left(0,0\right)p\left(1,0|0,0\right)\\ +p(0,1)p(1,1|0,1)+p(1,0)p(0,0|1,0)+p(1,1)p(0,1|1,1)]. \end{array}$$

The reason for the subtraction of (14) in (17) is to take into account removing the cases wherein NU can decode its data even though the SIC fails. To continue the derivation of the equations,

(18) $$\begin{array}{c} p(0,1|1,1)=p(0,0|1,0)\\ p(0,1|1,1)=p(1,0|0,0), \end{array}$$

(19) $$\begin{array}{c} p\left(0,0|1,0\right)=\frac{1}{\sqrt{N_{0}\pi }}\int_{\frac{2p_{f}+p_{n}}{2}h\sqrt{E_{b}}}^{\infty }e^{\frac{-{\left(y-p_{n}h\sqrt{E_{b}}\right)}^{2}}{N_{0}}}dy, \end{array}$$

(20) $$z=\frac{y-p_{n}h\sqrt{E_{b}}}{\sqrt{N_{0}}},$$

(21) $$\begin{array}{cc} p(0,0|1,0) & =\frac{1}{\sqrt{\pi }}\int_{\frac{2p_{f}-p_{n}}{2}h\sqrt{\frac{E_{b}}{N_{0}}}}^{\infty }e^{-z^{2}}dz\\ \; & =\frac{1}{2}erfc\left(\frac{2p_{f}-p_{n}}{2}h\sqrt{\frac{E_{b}}{N_{0}}}\right), \end{array}$$

p(0,1|1,1) is derived below:

(22) $$\begin{array}{c} p\left(0,1|1,1\right)=\frac{1}{\sqrt{N_{0}\pi }}\int_{\frac{h\sqrt{E_{b}}}{2}}^{\frac{p_{n}h\sqrt{E_{b}}}{2}}e^{\frac{-{\left(y-h\sqrt{E_{b}}\right)}^{2}}{N_{0}}}dy, \end{array}$$

(23) $$z=\frac{y-h\sqrt{E_{b}}}{\sqrt{N_{0}}},$$

(24) $$p\left(0,1|1,1\right)=\frac{1}{\sqrt{\pi }}\int_{-\frac{1}{2}h\sqrt{\frac{E_{b}}{N_{0}}}}^{\frac{p_{n}h\sqrt{E_{b}}-2h\sqrt{E_{b}}}{2\sqrt{N_{0}}}}e^{-z^{2}}dz.$$

Then, the final equation for the failed SIC is

(25) $$\begin{array}{c} p\left(e|failedSIC\right)=\frac{1}{4}\left(erfc\left(0.5h\sqrt{\frac{E_{b}}{N_{0}}}\right)+erfc\left(\left(p_{f}-\frac{1}{2}\right)h\sqrt{\frac{E_{b}}{N_{0}}}\right)\right)\\ -\frac{1}{4}\left(erfc\left(\frac{2p_{f}-p_{n}}{2}h\sqrt{\frac{E_{b}}{N_{0}}}\right)+\frac{1}{\sqrt{\pi }}\int_{-\frac{1}{2}h\sqrt{\frac{E_{b}}{N_{0}}}}^{\frac{p_{n}h\sqrt{E_{b}}-2h\sqrt{E_{b}}}{2\sqrt{N_{0}}}}e^{-z^{2}}dz\right). \end{array}$$

Finally, the BER of NU with imperfect SIC is

(26) $$\begin{array}{c} p(e)=p(e|SIC)+p(e|failedSIC). \end{array}$$

Using (14) and (26), the datasets for the training model can be generated. A detailed explanation for generating the datasets is provided in Section 3.1.

### 2.2. Factory Automation Scenario

**Figure 3:**
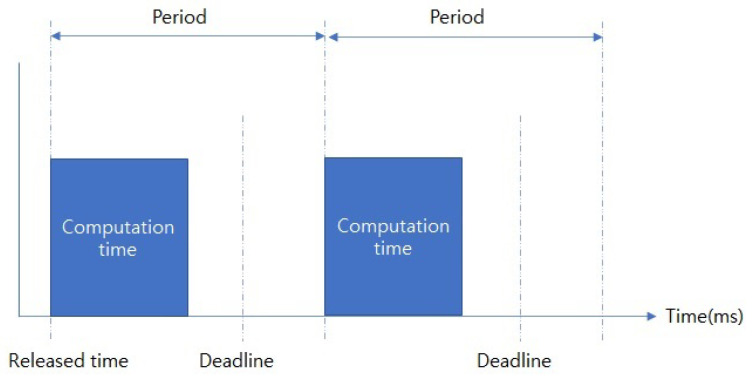
Message characteristic for factory automation.

In this study, we applied the VLC-NOMA system for the factory automation scenario [13,26]. The scenario is established based on time-division multiple access (TDMA) and superframe structure. The scenario includes periodic real-time messages sent by dedicated time slots considering beacon enable mode. The message characteristics are described in Figure 3.

(27) c≤d≤p

Computation time *c* is less or equal to deadline *d*, *d* is less or equal to period *p*. After the released time, a message will be generated with cycle *p* and should be transmitted by *d*. Every message for the VLC nodes has the characteristic mentioned above. Therefore, the message transmissions should be managed to be finished within their deadlines.

**Figure 4:**
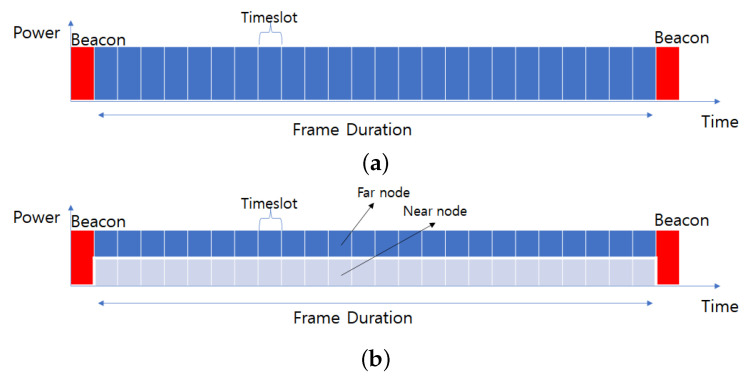
(**a**) OMA frame structure; (**b**) NOMA frame structure.

Figure 4 shows frame structures for VLC-OMA and VLC-NOMA systems. VLC-OMA and VLC-NOMA systems have the same frame duration consisting of 25 timeslots. The beacon starts at the beginning of a frame. Each timeslot is 10 ms. The difference between VLC-OMA and VLC-NOMA is whether to superpose signals or not. Figure 4a shows that the VLC-OMA system transfers signal for a node in a timeslot. Instead, shown in Figure 4b, the VLC-NOMA system can transfer two signals for two nodes in a timeslot by allocating different power levels.

**Figure 5:**
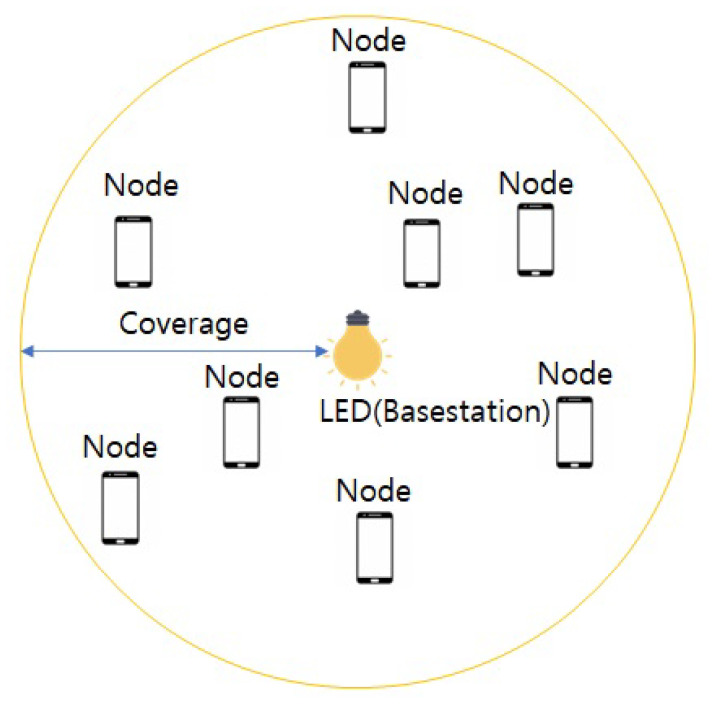
Deployed nodes and LED for factory automation.

Arrangements of nodes and LED are described in Figure 5. There is one LED, as a base station, and multiple nodes. Messages for the nodes are generated periodically from the LED. The messages have the characteristics described in Figure 3. Although a previous work [1] considered one message in one timeslot, the proposed system in this study considers two messages in one timeslot as it adopts the NOMA system with the superposition coding. Next, Section 3 describes resource allocation schemes for the VLC-NOMA system in the factory automation scenario.

## 3. Proposed Resource Allocation Schemes

In this section, to improve the reliability of the downlink NOMA system with VLC, DBPA for power allocation and PBUP for user-pairing are described.

### 3.1. DNN-Based Power Allocation

In this subsection, DBPA for power allocation between NU and PU is described. A multi-layer perceptron (MLP) is adopted for power allocation between NU and FU. An MLP is a class of feedforward artificial neural networks. It comprises an input layer, one or more hidden layers, and an output layer. The proposed MLP model comprises two hidden layers. A detailed model description is shown in Table 2. The input layer consists of three nodes—that is, the channel gains between NU and FU from the transmitter, and the noise level. The channel gains are calculated by (1), which considers the distance and angles between the LED and node. Therefore, channel gains can be considered representative of parameters such as the distance and the angles. Then, we assume noise levels $N_{0}$ are the same for all nodes. The output layer consists of one node, which is the power allocation factor for NU, $p_{n}$. The power allocation factor for FU, $p_{f}$, is not included in the output because we can obtain it by subtracting $p_{n}$ from 1. This implies that the power factors for both users can be expressed as $1=p_{n}+p_{f}$. Additionally, the rectified linear unit (ReLU) was adopted as the activation function. It has two additional major benefits: sparsity and reduced likelihood of vanishing gradient. The activation function of this model, ReLU *f*, is defined as follows:

(28) $$f\left(x\right)=\left\{\begin{array}{cc} 0 & \mathrm{if}\;x\;<\;0,\\ x & \mathrm{if}\;x\;\geq \;0. \end{array}\right.$$

This model consists of two hidden layers; its equation is expressed as

(29) $$Y=f(f\left(f\left(XW_{1}^{T}\right)W_{2}^{T}\right)W_{3}^{T}).$$

*X* is the input vector consisting of the channel gains between users and the transmitter and the noise level, *W* is the weight vector of each layer, and *T* is the transpose. The output *Y* is the power ratio for NU, $p_{n}$. Using the regression output of the model, the power ratio between NU and FU can be obtained.

Based on (14) and (26), the average BER of both NU and FU with imperfect SIC can be calculated. As the study focuses on improving reliability, the minimum point of the average BER for NU and FU can be determined by changing the power allocation factor. Therefore, the power allocation factor that results in the minimum average BER can be used as a dataset compared with the model’s output. The equation of average BER on power ratio for NU and FU is as follows:

(30) $$\begin{array}{c} p\left(e|h_{n},h_{f},p_{n}\right)=\frac{1}{8}[\left(erfc\left(0.5h_{f}\sqrt{\frac{E_{b}}{N_{0}}}\right)+erfc\left(\left(0.5-p_{n}\right)h_{f}\sqrt{\frac{E_{b}}{N_{0}}}\right)\right),\\ +\left(2-0.5\left(erfc\left(0.5h_{n}\sqrt{\frac{E_{b}}{N_{0}}}\right)+erfc\left(\left(0.5-p_{n}\right)h_{n}\sqrt{\frac{E_{b}}{N_{0}}}\right)\right)\right)erfc\left(\frac{p_{n}h_{n}}{2}\sqrt{\frac{E_{b}}{N_{0}}}\right)\\ +\left(erfc\left(0.5h_{n}\sqrt{\frac{E_{b}}{N_{0}}}\right)+erfc\left(\left(0.5-p_{n}\right)h_{n}\sqrt{\frac{E_{b}}{N_{0}}}\right)\right)-(erfc\left(\left(1-1.5p_{n}\right)h_{n}\sqrt{\frac{E_{b}}{N_{0}}}\right)\\ +\frac{1}{\sqrt{\pi }}\int_{-\frac{1}{2}h_{n}\sqrt{\frac{E_{b}}{N_{0}}}}^{\frac{p_{n}h_{n}\sqrt{E_{b}}-2h_{n}\sqrt{E_{b}}}{2\sqrt{N_{0}}}}e^{-z^{2}}dz)]. \end{array}$$

**Figure 6:**
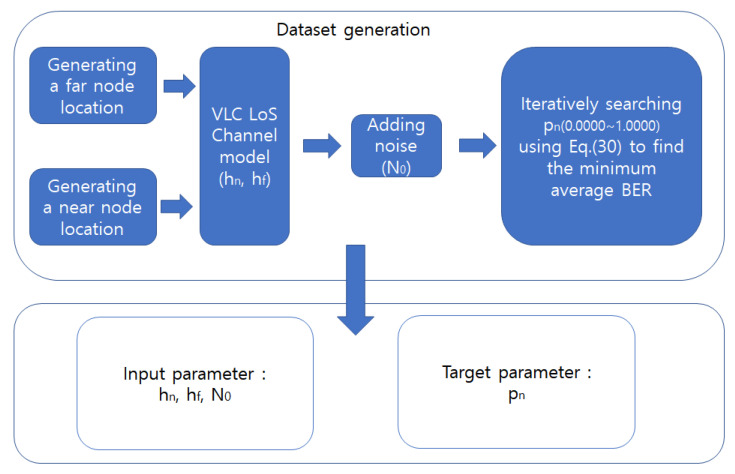
Dataset generation.

$h_{n}$ and $h_{f}$ are the channel gains for NU and FU, respectively. $P_{n}$ is the power allocation ratio for NU. The minimum average BER can be determined by iteratively changing $p_{n}$ from zero to one. However, finding the minimum average BER iteratively takes a lot of time in a real-time situation. Instead, an iterative method can be used to create datasets for training the model. The power ratio, which can make the minimum average BER, can be used as a training dataset. As shown in Figure 6, datasets for training a DNN model for DBPA are generated. First, the locations of FU and NU are randomly generated. Based on the locations, the channel states for the nodes are calculated based on (1). Then, the channel states $h_{n}$ and $h_{f}$, and noise $N_{0}$ are used as the inputs for (30) to calculate the average BER. The power ratio for near node $p_{n}$ is searched from 0.0 to 1.0 by step 0.0001. By iteratively searching, the minimum average BER is found as a target value to train the DNN model for DBPA.

The power ratio iteratively found is compared with the output of the proposed DNN model for updating the weights. To update the weights of the model, the adaptive moment estimation (ADAM) optimizer was adopted [27]. ADAM adjusts the learning rate by estimations of the fist and second moments of the gradient and has the advantages that the magnitudes of parameter updates are immutable regardless of gradient rescaling, its step sizes are approximately bounded by the step size hyperparameter, it does not require a stationary objective, it works with sparse gradients, and it naturally performs a form of step size annealing.

### 3.2. Proposed User Pairing Scheme

In this subsection, PBUP is proposed for user-pairing considering priorities in the downlink VLC-NOMA system. We assume two users in a user-pair.

Algorithm 1 explains how the proposed PBUP works. If there are more than two released nodes, the node with the earliest deadline will have the highest priority, and its channel gain will be used to find another node to be user-paired. The other node to be user-paired will be decided by the difference between the channel gains. Every released node is compared with the node with the highest priority by channel gains. Then, the one with the largest different channel gain from the node with the highest priority will be user-paired with the node with the highest priority. Then, the node with a higher channel gain will be considered NU. Using DBPA, the power ratio between NU and FU will be decided. After deciding on the user-pair and power allocation, the transmission will proceed in one timeslot.
**Algorithm 1:** Proposed user pairing scheme
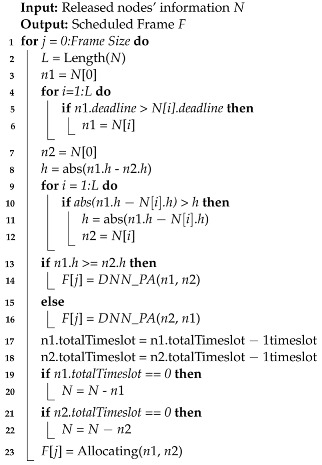


## 4. Evaluation and Analysis

In this section, to evaluate DBPA in the downlink VLC-NOMA, simulation and numerical results are discussed. In the first subsection, the BER performance of the downlink VLC-NOMA system is evaluated to create datasets correctly. In the second subsection, DBPA is evaluated in terms of the average BER compared with other power allocation algorithms.

### 4.1. Evaluation of the System Model

In this subsection, the proposed downlink VLC-NOMA system is evaluated in terms of imperfect SIC by numerical results and simulation results for BER performance. Table 3 describes parameters for the evaluation, where Python was used as a simulation tool. As described in Figure 1, the system is made up of two users as receivers and an LED as a transmitter. To evaluate the system, the average BER for both users is calculated using (30) with regard to the signal-to-noise ratio (SNR).

**Figure 7:**
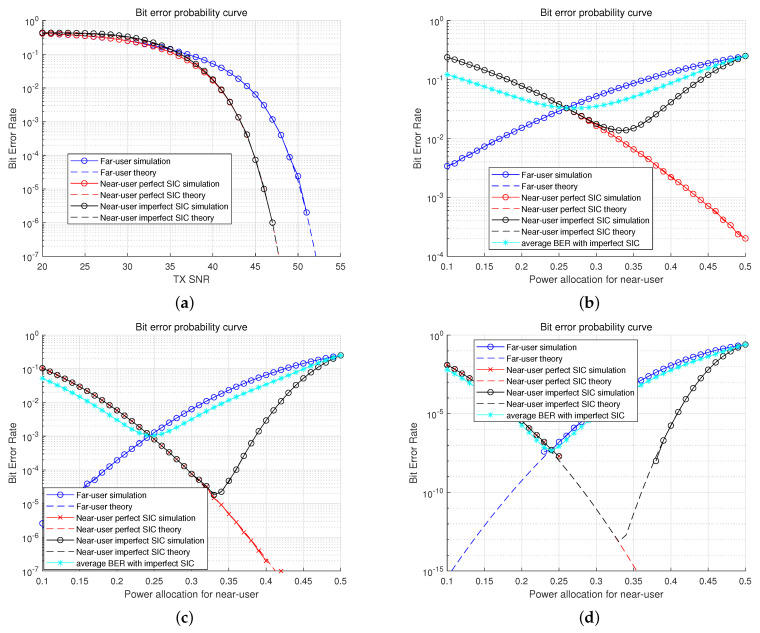
BER comparisons. (**a**) BER comparison. (**b**) BER comparison (Transmit SNR 40 dB). (**c**) BER comparison (Transmit SNR 45 dB). (**d**) BER comparison (Transmit SNR 50 dB).

Figure 7a shows the evaluation results of FU and NU in the perfect and imperfect SICs, varying the transmit SNR. The trends show no difference between the numerical results and the simulation results. When the transmit SNR is approximately 35 dB, the far and NUs in the perfect SIC show a similar performance. Despite assuming the perfect SIC, the BER performance of NU is not lower than that of FU. From 35 dB, the BER result of NU finally starts showing lower than that of FU. The BER performance of an NU with imperfect SIC is worse until 34 dB than the others because decoding FU’s signal on the side of NU for SIC can fail. If NU fails to decode the signal of FU, NU cannot SIC FU’s signal and decode its own signal. However, increasing transmit SNR causes lower decoding failures for SIC and enhances the BER performance for NU with imperfect SIC. The BER performances for NU between imperfect SIC and perfect SIC start overlying at 4 dB. The success rate of decoding FU’s signal on the side of NU affects the BER performance of NU with imperfect SIC.

Figure 7b shows the evaluation results of FU and NU varying the power allocation ratio at 40 dB transmit SNR. The BER performances of NU with perfect SIC and FU meet at the 0.26 power ratio for NU. The average BER performance between NU with imperfect SIC and FU is the lowest at this point, and the results worsen beyond this point because the failure rate for cancellation of FU’s signal increases.

Figure 7c describes that the average BER performance is the lowest at the 0.24 power ratio in the transmit SNR 45.

Figure 7d describes that the BER trends are similar to Figure 7b,c.

According to Figure 7, the power ratio with the lowest point of average BER with imperfect SIC can be the target dataset to train DBPA.

### 4.2. Evaluation of the Proposed DNN-Based Power Allocation

In this subsection, DBPA is evaluated in terms of the loss rate and average BER by numerical results. As the model aims to obtain the minimum average BER by changing the power allocation ratio, the output of the model is compared to the minimum point of the average BER for learning and will be used as a power allocation ratio for NU.

**Figure 8:**
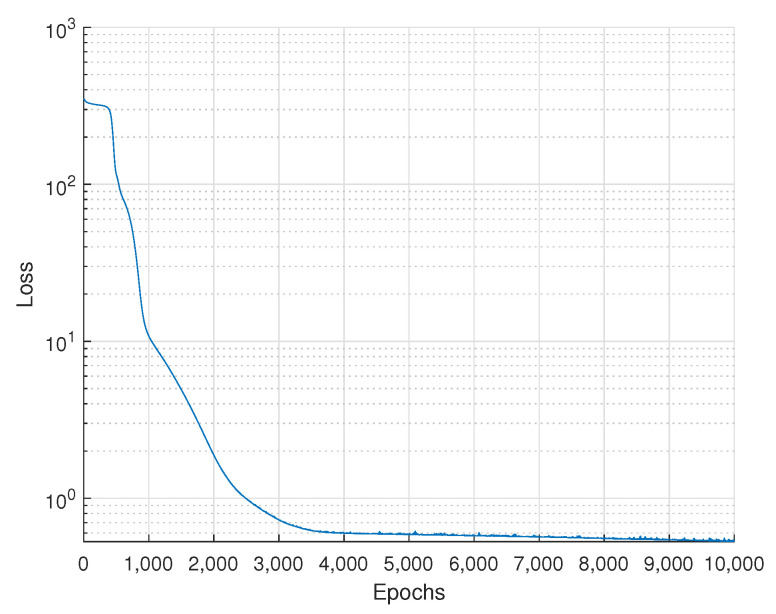
Loss for learning.

**Figure 9:**
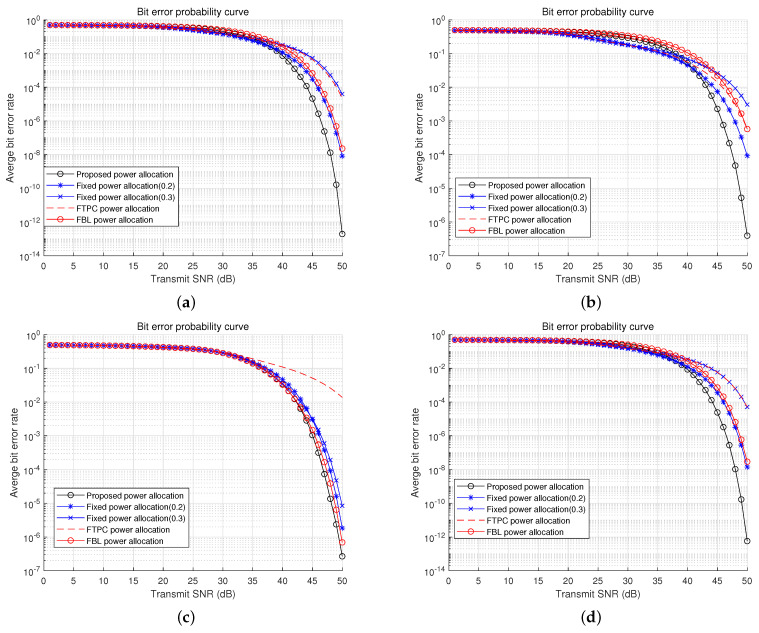
BER comparisons (**a**) BER comparison ($h_{f}$= 0.0251, $h_{n}$= 0.4010). (**b**) BER comparison ($h_{f}$= 0.0402, $h_{n}$= 0.3675). (**c**) BER comparison ($h_{f}$= 0.0445, $h_{n}$= 0.1002). (**d**) BER comparison ($h_{f}$= 0.0395, $h_{n}$= 0.3290).

In Figure 8, the loss of learning is shown along epochs. To create datasets, the locations of FUs, NUs, and LEDs are set as listed in Table 4, and the other simulation parameters are the same as listed in Table 3. The number of epochs for learning was 10,000 and the batch size was 64. We can see that the loss reduces as learning progresses. Figure 9 shows the average BER comparisons between power allocation algorithms with the proposed DNN model. The numerical results based on (30) are compared among power allocation algorithms, including the proposed algorithm. The channel gains are decided by varying the locations of nodes. As the locations of nodes are randomly chosen, the channel gains are randomly decided. According to the simulation results, the range of $p_{n}$ was from 0.002 to 0.482. The users’ BERs are evaluated by power allocation algorithms such as fixed power allocations, fractional transmit power control (FTPC) [28,29,30], finite blocklength (FBL)-based power allocation [31], and DBPA. The average BER performances are decided by channel gains and power allocation algorithms. They show that DBPA has the lowest BER performance among all algorithms.

### 4.3. Evaluation of the Proposed User-Pairing

In this subsection, the simulation results of scheduling by OMA with deadline monotonic scheduling (DMS), NOMA with the next-largest-difference-based user pairing algorithm (NLUPA) [30], and NOMA with PBUP are dealt with. Simulation parameters are shown in Table 5. In the case of NLUPA, the transmission order is as the following example. For example, if there are four nodes released, the first user pair, which consists of the node with the largest channel gain and the node with the lowest channel gain, will be transmitted first, and the next transmission is on the next user pair that consists of the node with the second largest channel gain and the node with the second lowest channel gain. Except for OMA, DBPA is adopted for NOMA systems as the power allocation scheme. In this simulation, 10 nodes are deployed at locations shown in Table 5. Released time means when the messages for nodes are released. Computing time means how many timeslots are required for transmission. Deadline means when the transmission should be performed by after being released. Period means the time when messages appear periodically. Simulation time is 10,000 ms, which means 1000 timeslots.

As shown in Table 6, missed deadlines are evaluated by simulation. Missed deadline means that a transmission is not finished by the deadline. By comparisons among OMA with DMS and NOMA with NLUPA, the simulation results show that PBUP has fewer missed deadlines than the others. The OMA system handles only one transmission during a timeslot; the NOMA system handles a maximum of two transmissions during a timeslot using superposition coding. Comparing PBUP with NLUPA, PBUP considers the deadline-based priority; instead, NLUPA only considers user-pairing. Therefore, PBUP has fewer missed deadlines than the others.

The packet delivery ratio (PDR) means transmissions succeed without packet errors and missed deadlines. The evaluation results are shown in Table 7. The evaluation proceeded with OMA with DMS, NOMA with PBUP, and NOMA with NLUPA. The NOMA systems adopted the DBPA as the power allocation scheme between NU and FU. At transmitted SNR 40 dB, OMA with DMS showed higher PDR than the others, because NOMA systems should divide transmitted power between NU and FU. It causes higher packet errors than OMA. However, from 41 dB, the NOMA systems start improving PDR until 49 dB. From 49 dB, the saturation starts because of missed deadlines and no packet errors. No more reduction of packet errors and missed deadlines are generated from 49 dB. Table 7 shows that the NOMA system with PBUP and DBPA outperforms the others.

## 5. Conclusions

In this study, a downlink VLC-NOMA system was investigated in a factory automation scenario. A DNN-based power-allocation scheme (DBPA) and a priority-based user-pairing (PBUP) scheme were proposed to improve the reliability of the system. In terms of the minimal average BER, DBPA was compared with fixed power allocation, FTPC, and FBL-based algorithms. DBPA exhibited the lowest average BER among the power allocation algorithms. Thus, DBPA can be applied to VLC-NOMA systems to improve reliability. PBUP in the VLC-NOMA system was compared with DMS in the VLC-OMA system and NLUPA in the VLC-NOMA system. The evaluation results show that the VLC-NOMA system with the proposed DBPA and PBUP outperforms VLC-OMA with DMS and VLC-NOMA with NLUPA in the factory automation scenario. Future work will consider multiple LEDs to handle multiple nodes to improve reliability in factory automation scenarios.

## Figures and Tables

**Table 1:** Table for notations.

Variable	Definition
*m*	Lambertian emission order
*A*	area of PD
*T*	optical filter gain
Φ	angle of irradiance
ϕ	angle of incidence
$\Phi_{1/2}$	Semi-angle of LED
$p_{f}$	transmission Power ratio for FU
$p_{n}$	transmission Power ratio for NU
$h_{f}$	channel gain between FU and LED
$h_{n}$	channel gain between NU and LED
$y_{f}$	received signal for FU
$y_{n}$	received signal for NU
$x_{f}$	transmitted signal for FU
$x_{n}$	transmitted signal for NU
erfc	error function
$E_{b}$	energy per bit
$N_{0}$	noise power

**Table 2:** Proposed MLP model structure.

Input layers	Channel states for NU and FU, $h_{n}$ and $h_{f}$, and noise level $N_{0}$
Output layers	Power allocation ratio for NU, $p_{n}$
Structure of network	3 × 8 × 8 × 1

**Table 3:** Simulation parameters.

LED axis (x, y, z)	5 m, 5 m, 2.5 m
NU axis (x, y, z)	5 m, 6 m, 1.5 m
FU axis (x, y, z)	6 m, 6 m, 1.5 m
Transmitted Power	60 W
Power ratio for NU	0.3
Power ratio for FU	0.7
Semi-angle of LED	60°
Optical-filter gain	1
Area of PD	$10^{-4}$ m${}^{2}$
Refractive index of PD lens	1.5
PD’s field of view	60°

**Table 4:** Users’ positions.

LED axis (x, y, z)	5 m, 5 m, 2.5 m
NUs’ axes (x, y, z)	(4.5~5.5) m, (4.5~5.5) m, 1.5 m
FUs’ axes (x, y, z)	(3.8~4.5,5.5~6.2) m, (3.8~4.5, 5.5~6.2) m, 1.5 m

**Table 5:** Scheduling parameters.

Number of nodes	10
X-axis of nodes	4∼6 m
Y-axis of nodes	4∼6 m
Z-axis of nodes	1.5 m
Released times	10∼100 ms
Computing times	10∼50 ms
Deadlines	10∼100 ms
Periods	10∼200 ms
Superframe size	250 ms
Simulation time	100,000 ms
Bits per packet	32

**Table 6:** Missed deadline comparisons.

	OMA with DMS	NOMA with PBUP	NOMA with NLUPA
Missed deadlines	4844	1910	2320

**Table 7:** Packet delivery ratio.

TX SNR (dB)	40	41	42	43	44	45	46	47	48	49	50
OMA with DMS (%)	34.99	35.21	35.26	35.3	35.3	35.3	35.3	35.3	35.3	35.3	35.3
NOMA with PBUP (%)	34.23	42.34	49.14	54.67	59.64	64.11	68.06	71.9	73.47	74.49	74.49
NOMA with NLUPA (%)	28.49	36.6	43.34	48.7	54.21	58.77	62.45	66.46	68.02	68.81	69.01

## Data Availability

Data sharing is being prepared.
